# A review of visual perception technology for intelligent fruit harvesting robots

**DOI:** 10.3389/fpls.2025.1646871

**Published:** 2025-08-19

**Authors:** Yikun Huang, Shuyan Xu, Hao Chen, Gang Li, Heng Dong, Jie Yu, Xi Zhang, Riqing Chen

**Affiliations:** 1 School of Future Technology, Fujian Agriculture and Forestry University, Fuzhou, China; 2 Concord University College, Fujian Normal University, Fuzhou, China; 3 Minnan University of Science and Technology, Quanzhou, China; 4 Fujian Key Lab of Agricultural Internet of Things Applications, Sanming University, Sanming, China

**Keywords:** intelligent fruit harvesting robots, agricultural robotics, visual perception, object detection, visual servoing, V-SLAM

## Abstract

With the development of smart agriculture, fruit picking robots have attracted widespread attention as one of the key technologies to improve agricultural productivity. Visual perception technology plays a crucial role in fruit picking robots, involving precise fruit identification, localization, and grasping operations. This paper reviews the research progress in the visual perception technology for fruit picking robots, focusing on key technologies such as camera types used in picking robots, object detection techniques, picking point recognition and localization, active vision, and visual servoing. First, the paper introduces the application characteristics and selection criteria of different camera types in the fruit picking process. Then, it analyzes how object detection techniques help robots accurately recognize fruits and achieve efficient fruit classification. Next, it discusses the picking point recognition and localization technologies, including vision-based 3D reconstruction and depth sensing methods. Subsequently, it elaborates on the adaptability of active vision technology in dynamic environments and how visual servoing technology achieves precise localization. Additionally, the review explores robot mobility perception technologies, focusing on V-SLAM, mobile path planning, and task scheduling. These technologies enhance harvesting efficiency across the entire orchard and facilitate better collaboration among multiple robots. Finally, the paper summarizes the challenges in current research and the future development trends, aiming to provide references for the optimization and promotion of fruit picking robot technology.

## 1 Introduction

With the continuous growth of the global population, agricultural production is facing increasingly severe challenges. Rising labor costs, increased labor intensity for farmers, and low agricultural productivity have become key factors limiting the development of modern agriculture (Zhang et al., 2024). To address these issues, agricultural automation technologies have emerged, with intelligent harvesting robots receiving widespread attention and research as an efficient and automated solution (Chunjiang et al., 2023). With the rapid advancements in artificial intelligence, robotics, and computer vision technologies, fruit harvesting robots have gradually become a focal point of research.

**Figure 1:**
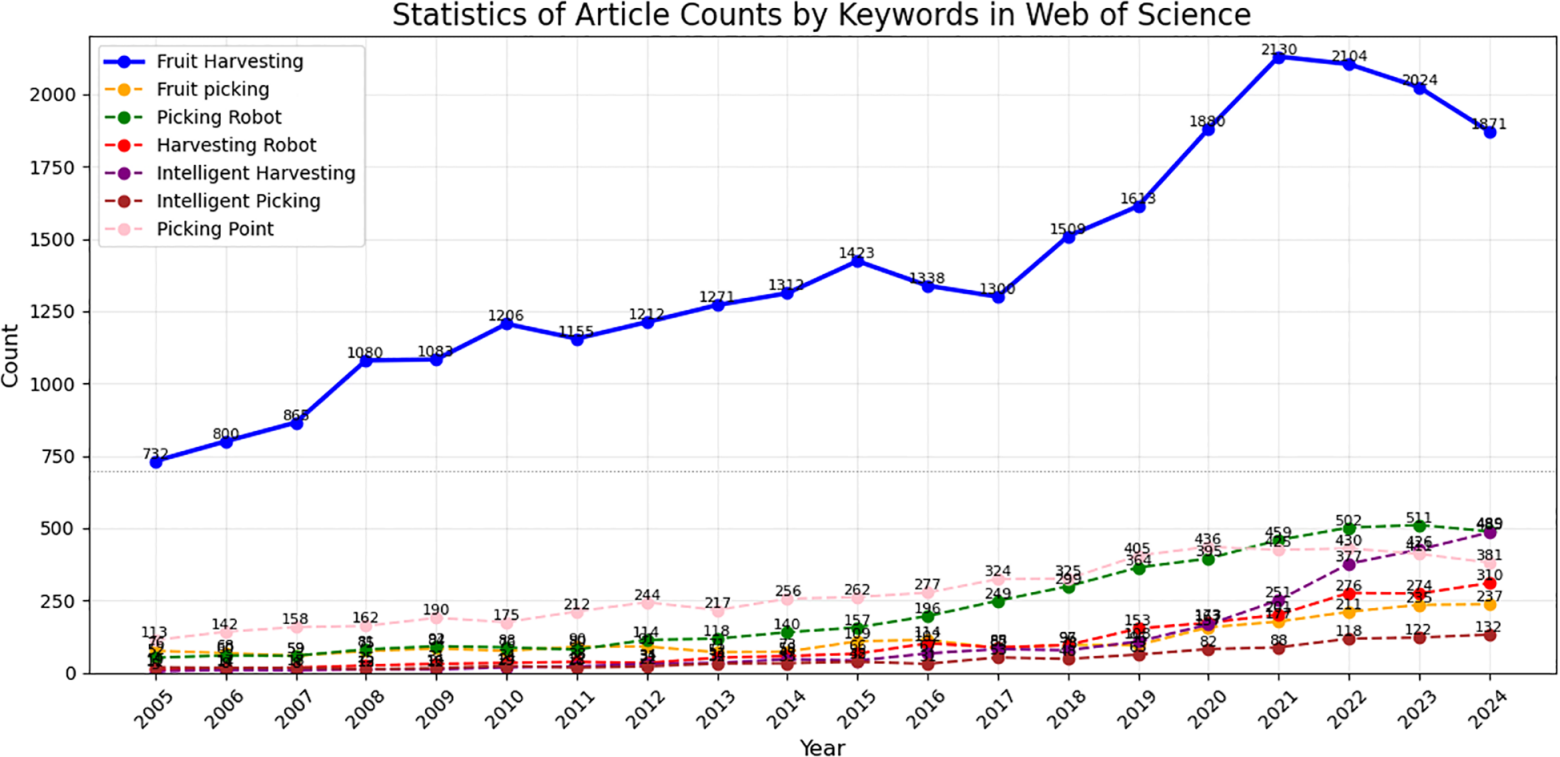
Statistics of article counts by keywords in web of science.

In order to provide a comprehensive understanding of the research trends in this field, we conducted a statistical analysis of related research articles from 2005 to 2024 based on the Web of Science database, as show in **Figure 1**. The results show a significant increase in the number of publications in the field of “Fruit Harvesting,” rising from 732 articles in 2005 to 2130 in 2024. This indicates that, with the rapid development of smart agriculture technologies, the research interest in this field has continued to grow, with visual perception and robotics technologies gradually becoming the core focus of research.

### 1.1 Development status of intelligent fruit harvesting robots

In 1968, the United States pioneered the study of fruit harvesting using mechanical or pneumatic vibration methods. Although these methods could perform basic harvesting tasks, vibration and pneumatics often caused significant damage to the fruit (Schertz and Brown, 1968). With the development of computer and control technologies, agricultural robots began to be applied in tasks such as harvesting, spraying, and weeding from the 1990s onward, assisted by computer vision. In particular, some robotic arms were able to simulate manual harvesting actions. However, due to the limitations of robot and sensor technologies at the time, automated harvesting robots still faced challenges such as high costs, low precision, and limited application scenarios. With the rapid development of Industry 4.0, advancements in artificial intelligence, the Internet of Things, and big data analysis have greatly propelled the progress of agricultural harvesting robots, especially in the precision of perception, autonomous decision-making, control, and execution (Oliveira et al., 2021). In particular, the continuous innovation of visual perception systems has provided harvesting robots with more powerful sensing capabilities. Modern intelligent fruit harvesting robots are now able to obtain real-time environmental information through devices such as cameras, LiDAR, and depth sensors, and identify the type, location, and status of fruits using image processing and pattern recognition technologies.

**Figure 2:**
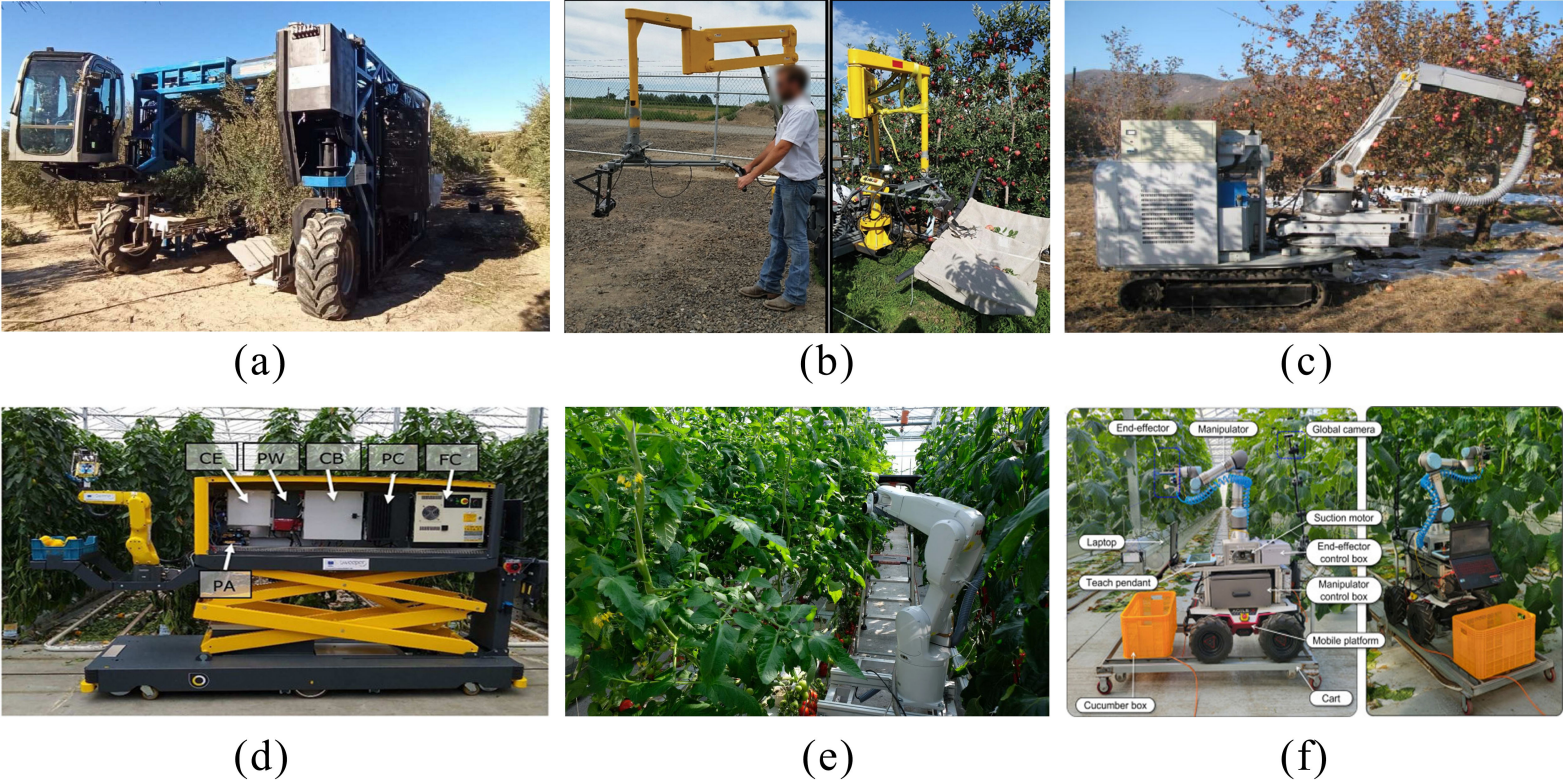
Various types of harvesting robots. **(a)** Olive Shaking Bulk Harvesting Equipment (Sola-Guirado et al., 2023), **(b)** Apple Vibration Harvesting Robot (De Kleine and Karkee, 2015), **(c)** Apple Selective Harvesting Large-Scale Equipment (Jia et al., 2020), **(d)** Sweet Pepper Harvesting Robot (Arad et al., 2020), **(e)** Tomato Harvesting Robot (Rapado-Rincón et al., 2023), **(f)** Cucumber harvesting robot (Park et al., 2023).

Harvesting robots can be divided into bulk harvesting robots and selective harvesting robots (Zhou et al., 2022). As shown in **Figures 2a-c**, bulk harvesting robots are typically large and perform one-time harvesting by applying vibration or forced separation to the fruit trees. Examples include apple harvesting by vibrating branches (De Kleine and Karkee, 2015), cherry harvesting by vibration (Zhou et al., 2016), and bulk grape harvesting for industrial use (Yan et al., 2023). Although bulk harvesting methods are efficient, they cause significant damage to the fruits and are difficult to distinguish based on ripeness, making them suitable only for industrial fruit, not for those intended for market sales.

Selective harvesting robots typically install the end effector on a robotic arm and use computer vision to identify ripe fruits, guiding the robotic arm and end effector to perform the harvesting task, as shown in **Figures 2d-f**. These devices are usually smaller in size and can move freely in agricultural environments. Since their harvesting method is the closest to human picking, they have already been applied in harvesting fruits such as apples (Jia et al., 2020), peppers (Arad et al., 2020), tomatoes (Rapado-Rincón et al., 2023), yellow peaches (Wang et al., 2023), and strawberries (Tafuro et al., 2022). Although selective harvesting robots have lower work efficiency, they support batch harvesting and effectively reduce fruit damage, thus preserving the commercial value of the fruits. This method is considered the most likely to fully replace human harvesters, which has led to widespread attention to selective harvesting robots in both academia and industry (Sanders, 2005).

### 1.2 The importance of visual perception technology in fruit harvesting

Visual perception technology plays a pivotal role in intelligent fruit harvesting robots, serving as one of the core technologies enabling automated picking. It facilitates the accurate identification and localization of target fruits through image processing and object detection, ensuring the efficient and precise execution of harvesting tasks. The visual system must adapt to varying lighting conditions, diverse fruit types, and complex background environments. The application of deep learning, 3D reconstruction, and image segmentation techniques enhances its robustness and accuracy. Furthermore, visual perception supports dynamic decision-making for the robot, allowing real-time adjustments to harvesting strategies in response to fruit displacement or occlusion, thereby ensuring operational continuity and stability. With technological advancements, the introduction of visual servo systems and closed-loop control has further improved manipulation precision and minimized fruit damage.

Scholars have developed models for detecting picking points using image analysis and deep learning techniques to guide robotic manipulators in the intelligent harvesting of fruits such as pepper (Arad et al., 2020; Babellahi et al., 2020), tomato (Jun et al., 2021; Wu et al., 2021), apple (Jia et al., 2020; Li C. et al., 2023), and grape (Yan et al., 2023; Wang J. et al., 2024). In intricate field settings, factors such as fluctuating illumination, fruit overlap, variations in fruit maturity, accurate peduncle/stem recognition, and precise localization of the picking point significantly impact the operational efficiency and harvesting accuracy of robots. Concurrently, when fruits are occluded, determining the optimal viewing angle for observation and planning effective manipulator trajectories become critical challenges for enhancing harvesting performance. Therefore, a thorough examination of the latest advancements, existing challenges, and future trends in visual perception technology for fruit harvesting robots holds substantial academic significance and practical value for advancing the field.

## 2 Common camera types for harvesting robots

Efficient visual perception systems are fundamental to intelligent fruit harvesting robots, with cameras serving as core components whose performance is determined by sensor type and design. Driven by advancements in computer vision, deep learning, and sensor technology, traditional RGB cameras are increasingly being supplemented or replaced by various advanced sensors. Combining different sensors proves particularly effective in complex agricultural environments, significantly enhancing system robustness and accuracy. Common vision sensors include monocular cameras, binocular (stereo) cameras, RGB-D cameras and event cameras, each possessing distinct advantages and suitable application scenarios. **Table 1** presents a performance comparison of different types of cameras. The following will provide a detailed analysis of these camera types and explore their specific applications in fruit harvesting tasks.

**Table 1:** Comparison of different depth sensing technologies.

Technology	Monocular (Baeten et al., 2008)	Binocular stereo (Wu et al., 2021)	Structured light camera (Wang J. et al., 2024)	Time of flight camera (Li Z. et al., 2022)	Event camera (Rebecq et al., 2018)
Technology Principle	Captures 2D images using a single camera	Calculates depth information using the principle of disparity	Projects a light pattern and analyzes its deformation to acquire depth	Measures depth by the time difference of infrared light reflection	Pixel-level asynchronous brightness change detection
Depth Range	Estimated via algorithm	0.5–10 meters	0.2–5 meters	0.2–10 meters	Wide-range
Accuracy	Dependent on algorithm, low accuracy	Moderate	High	Moderate	High accuracy
Dynamic Scene Performance	Dependent on algorithm, performance is poor	Moderate	Good for static scenes, moderate for dynamic scenes	Excellent, suitable for dynamic scenes	Excellent, suitable for dynamic scenes
Advantages	Lowest cost, highest resolution	Provides direct depth information, moderate cost	High precision depth perception, suitable for near-field object recognition, good light adaptability	High precision, suitable for long-range, relatively stable	Ultra-low latency, ultra-high dynamic range and low power consumption
Disadvantages	Difficult to obtain depth information, highly affected by external light	Requires good scene texture, limited in poor lighting conditions	Sensitive to ambient light, higher cost	Affected by strong ambient light, accuracy decreases at longer distances	There is no texture information, so a dedicated algorithm is needed.
Providers	MOKOSE, HIKRobot, etc.	ZED, Intel RealSense D400 Series, etc.	Microsoft Kinect 1, Intel RealSense LR200, Orbbec Astra, etc.	Microsoft Kinect 2, Intel RealSense L515, SEERsense, etc.	Pixel-level asynchronous brightness change detection.

### 2.1 Monocular camera

**Figure 3:**
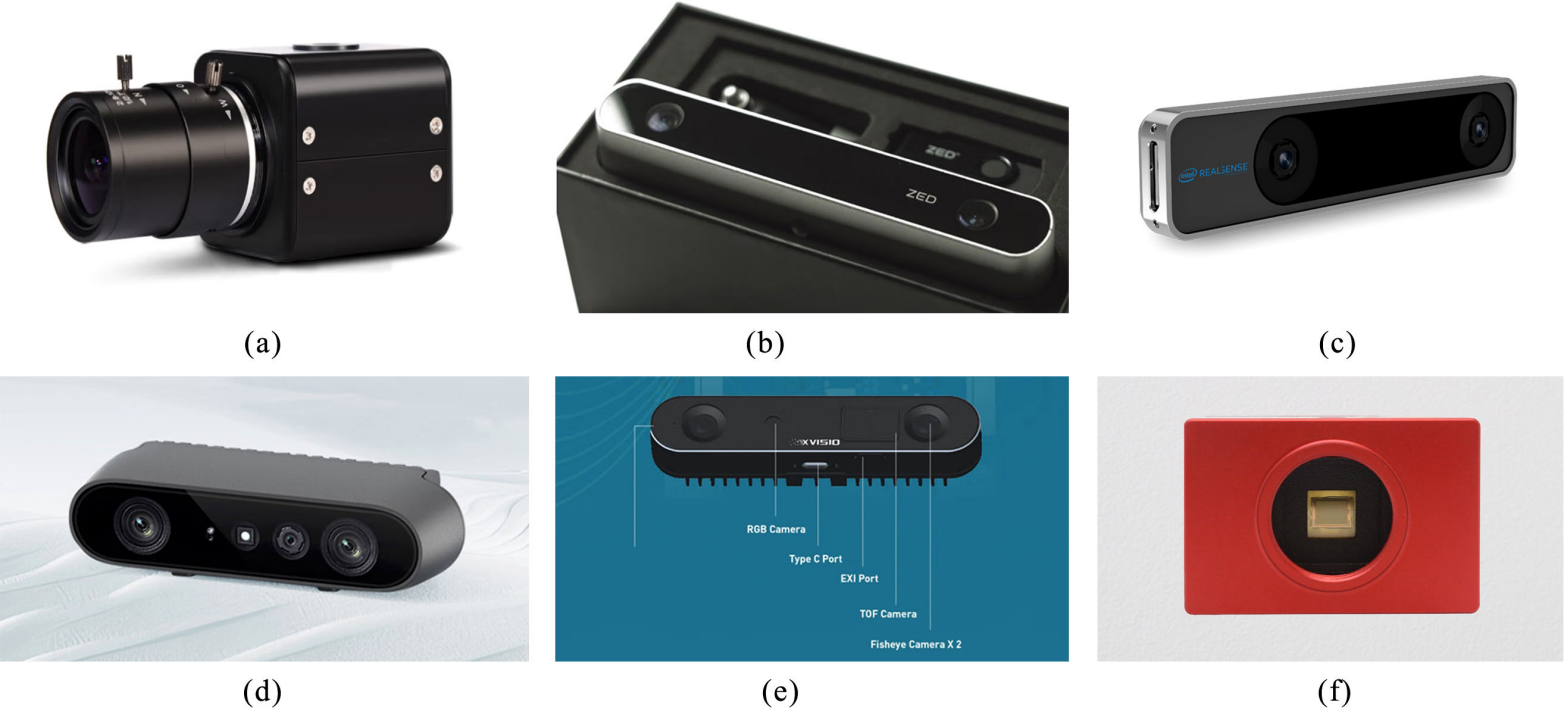
Representatives of cameras from different technology types. **(a)** MOKOSE monocular camera, **(b)** ZED stereo camera, **(c)** Intel T265 stereo camera. **(d)** ORBBEC structured light camera, **(e)** SEERSENSE ToF (Time of Flight) camera. **(f)** iniVation event camera.

Monocular cameras, capturing color images through a single lens, are widely utilized for image acquisition in deep learning applications due to their simple structure and low cost, as shown in **Figure 3a**. However, they are incapable of directly capturing depth information, providing only two-dimensional scene data, and are primarily used for tasks like object detection and yield estimation. To address this limitation, researchers employ deep learning and other methods to process monocular images and estimate fruit positions (Khan et al., 2020; Cheng et al., 2021; Yin et al., 2023). For instance, Yang et al. proposed a self-supervised monocular depth estimation network (Yang et al., 2020), while Ban et al. tackled depth estimation in defocused images using Markov random fields and geometric constraints (Ban et al., 2022). Despite these efforts, the lack of inherent depth data means monocular depth estimation still relies on computationally intensive methods and achieves limited accuracy. This challenge is particularly pronounced in unstructured agricultural scenes, where environmental complexity and indistinct object features further complicate depth estimation.

### 2.2 Stereo camera

Binocular cameras, as shown in **Figures 3b, c**, also referred to as stereo cameras, capture images of a scene using two lenses from different viewpoints. They compute object depth by leveraging the principle of parallax (Chao et al., 2023). By mimicking the human binocular vision system to acquire three-dimensional (3D) information, binocular cameras provide depth data more directly compared to monocular cameras. Consequently, they are widely adopted in agricultural robotics and automated harvesting scenarios due to their ability to deliver more accurate spatial localization in complex environments (Ling et al., 2019; Wu et al., 2021; Wen et al., 2022). However, binocular cameras also exhibit certain limitations. For instance, they exhibit a high dependency on texture features within the scene; depth estimation performance may degrade in texture-poor regions or under suboptimal lighting conditions. Furthermore, the hardware configuration of binocular cameras is inherently more complex than that of monocular cameras, demanding precise calibration and stringent synchronization between the two lenses.

### 2.3 RGB-D camera

To overcome the limitations of monocular and binocular cameras, RGB-D cameras have emerged as a solution. RGB-D cameras integrate an RGB color camera with a depth sensor, enabling simultaneous capture of color information and depth data from the scene, making them an increasingly popular choice for diverse applications. Beyond stereo vision, common methods for acquiring depth information with RGB-D cameras include structured light technology and Time of Flight (ToF) (Zhou et al., 2021) (as shown in **Figures 3d, e**). Structured light technology typically projects a known light pattern (e.g., stripes, dot arrays) onto object surfaces and captures the resulting deformation of this pattern using a camera to infer depth. Cameras employing this method offer high accuracy at close range and rapid depth acquisition, but depth measurement accuracy may decrease for objects with low reflectivity or lacking texture. Common structured light cameras include the Intel RealSense series, and the Intel RealSense D435 camera, valued for its compact size and high precision, is widely utilized in fruit harvesting tasks (Liu et al., 2024).

ToF calculates distance by emitting light pulses and measuring the time difference for the light to travel from the camera to the object and back. ToF can operate effectively under low-light conditions or significant illumination variations and provides rapid depth acquisition. However, its resolution is generally lower than that of structured light cameras, making it difficult to capture sufficiently detailed depth information in complex, close-range environments.

### 2.4 Event camera

In addition to conventional frame-based cameras, emerging vision sensors—such as event cameras—have demonstrated significant potential in agricultural applications, particularly in complex environments with high dynamic lighting conditions, as shown in **Figure 3f**. Unlike traditional cameras that capture entire images at fixed frame rates, event cameras operate using an asynchronous imaging mechanism that records data only when changes in pixel brightness occur (Gallego et al., 2021). Each event contains a timestamp, pixel location, and the polarity of brightness change, enabling ultra-high temporal resolution at the microsecond level, extremely low latency, and substantially reduced data redundancy. One of the most prominent advantages of event cameras is their exceptionally high dynamic range, often exceeding 120 dB, making them particularly effective in agricultural scenarios (Wan et al., 2024). For instance, event cameras can produce stable outputs under highly variable lighting conditions, such as when sunlight filters through foliage or when transitions occur rapidly between shaded and sunlit areas. Furthermore, their low power consumption and compact size make event cameras well-suited for integration into embedded systems and various field-deployed agricultural automation platforms.

In the context of precision agriculture, event cameras offer potential for a variety of tasks, including crop monitoring, where subtle structural changes in plants can be more effectively detected; real-time navigation of agricultural robots and UAVs in dynamically lit environments; and high-speed target detection (Gehrig and Scaramuzza, 2023), such as rapid identification of field animals, tracking of pest movements (Pohle-Fröhlich et al., 2024), or detection of fruit maturity status.

Compared to the aforementioned camera types, RGB-D cameras offer more stable depth perception in complex environments and exhibit reduced dependency on scene texture. They demonstrate superior performance in localization accuracy and computational efficiency (Zhou et al., 2022), making them well-suited for scenarios demanding high-precision depth information, such as agricultural robotics and autonomous driving. Given these advantages, RGB-D cameras have been successfully applied to the harvesting of various fruits (Yoshida et al., 2022).

### 2.5 Camera installation position

**Figure 4:**
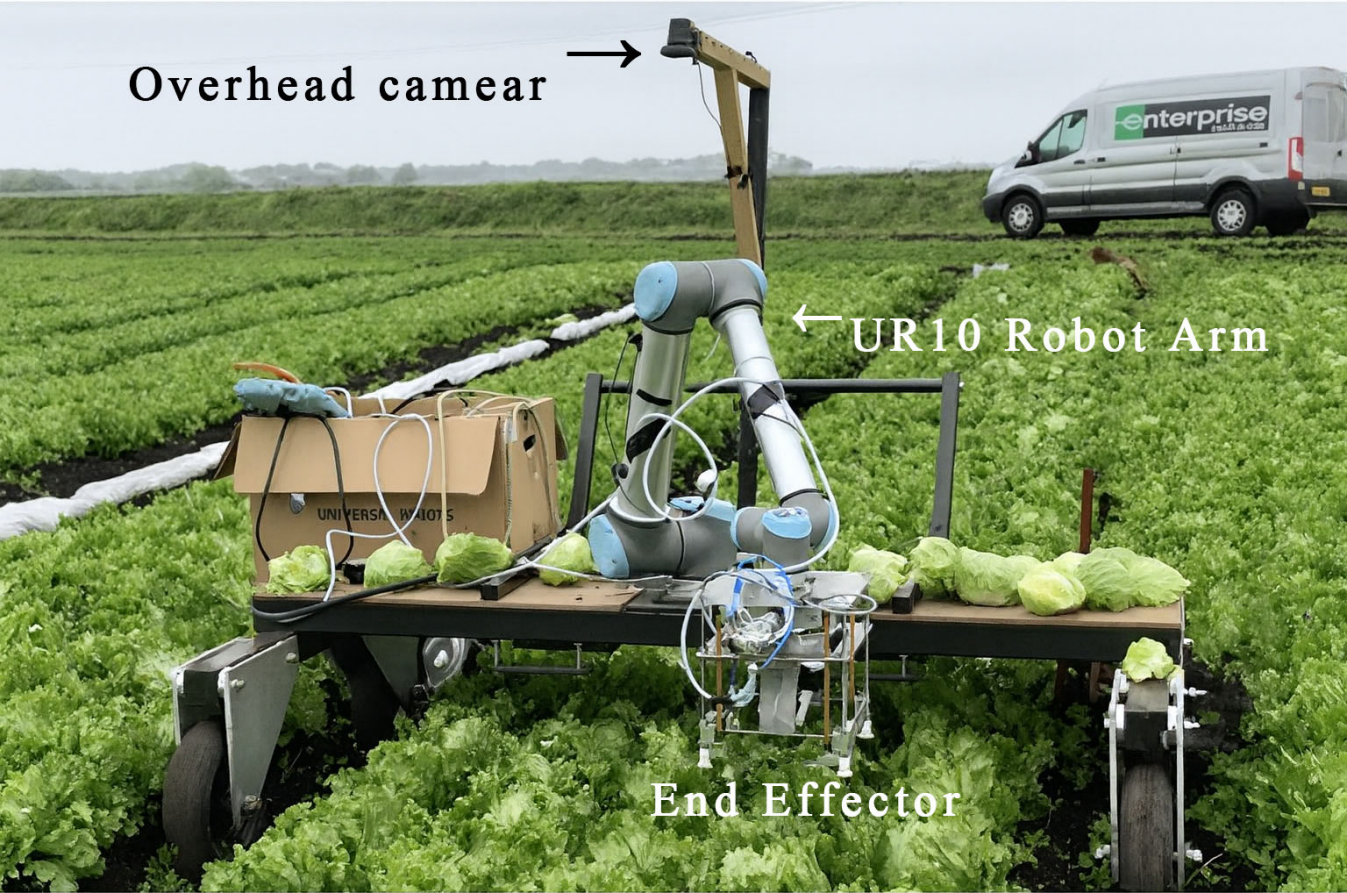
Eye-To-Hand robot.

**Figure 5:**
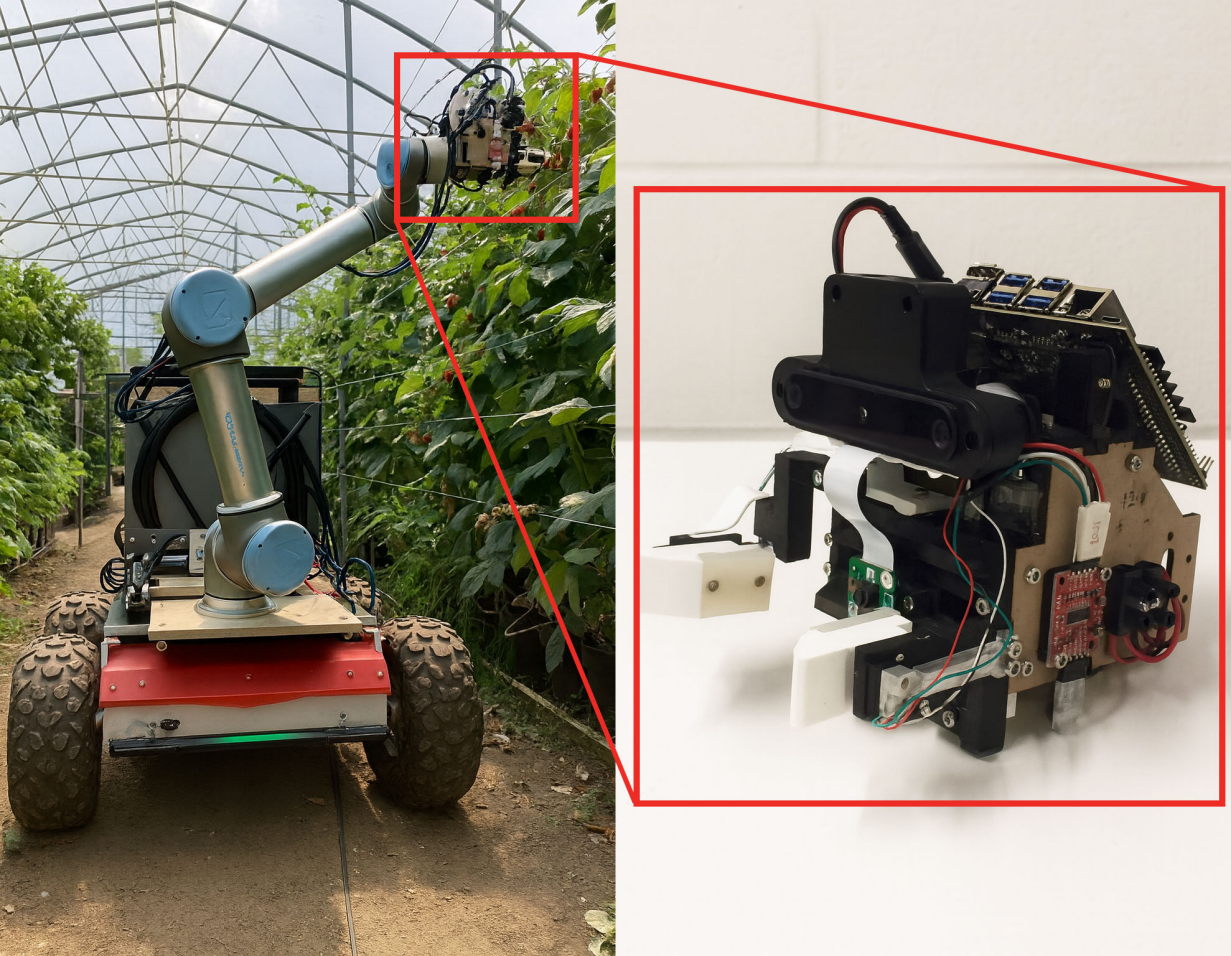
Eye-In-Hand robot.

The installation position of the camera directly determines the perception ability of the picking robot toward the fruits. A reasonable installation position can maximize the coverage of the visual perception system, enhance the ability to capture image details, and reduce the interference of external factors on recognition accuracy. Generally, the camera installation positions on a picking robot can be divided into Eye-To-Hand and Eye-In-Hand. Eye-To-Hand means the camera is installed at a fixed position on the robotic arm, typically on the robot’s base, workbench, or another location that does not change with the movement of the robotic arm. For example, Birrell et al. (2020) fixed the camera on a bracket in their lettuce harvesting system, as shown in **Figure 4**. This method provides stable visual information, but the fixed camera may fail to detect all the fruits due to occlusion. Eye-In-Hand refers to the camera being directly installed at the end of the robotic arm, where each movement of the arm directly affects the camera’s view. For example, Junge et al. (2023) installed an RGB-D camera at the end of the robotic arm in their strawberry picking robot design, with the camera moving along with the arm, as shown in **Figure 5**. This method is better at handling target localization and manipulation tasks in complex or confined spaces. However, its drawbacks include a larger computational load and a higher risk of the camera being damaged due to accidental collisions.

## 3 Object detection technology in fruit picking

Objective detection technology is the core technology in intelligent fruit harvesting (Xiao et al., 2024). Due to the vast variety of fruits, which exhibit significant variations in morphology, size, and color, object detection enables the training and optimization of recognition capabilities for different fruit types. Within harvesting tasks, object detection must first precisely locate fruit positions, assess maturity levels, evaluate occlusion conditions, and identify pickable points. Furthermore, it determines the picking sequence by analyzing fruit clustering before robotic arm execution, thereby enhancing harvesting efficiency and accuracy. Object detection techniques are typically categorized into traditional feature-based machine learning methods and deep learning-based approaches.

### 3.1 Traditional object detection technology

Traditional object detection methods primarily rely on the sliding window strategy and manual feature extraction. These include color features (such as threshold segmentation in HSV, Lab, and other color spaces), texture features (e.g., Gray-Level Co-occurrence Matrix - GLCM and Local Binary Patterns - LBP), and shape features (e.g., edge detection and Hough transform). Due to their distinctiveness and stability, color features are widely employed in fruit recognition, particularly in scenarios with simple backgrounds and high contrast between the fruit and its surroundings. For instance, Arefi et al. achieved an accuracy of 96.36% by combining features extracted from the RGB, HIS, and YIQ color spaces for tomato recognition (Arefi et al., 2011). Tian et al. utilized components of the HIS and LAB color spaces for tomato leaf segmentation (Tian et al., 2019), while Yamamoto et al. implemented target identification for strawberry harvesting through color threshold analysis, achieving a harvest rate of 67% (Yamamoto et al., 2014). In complex agricultural environments, OTSU adaptive thresholding is extensively applied to extract target fruit locations based on color differences (Wei et al., 2014; Lv et al., 2016). While color models prove effective in distinguishing fruits from backgrounds, their performance deteriorates significantly in complex backgrounds or when encountering objects with similar colors.

Morphological characteristics also hold significant importance in traditional methods. Features such as shape can be extracted through edge detection (e.g., the Canny operator) and contour detection (e.g., Hough transform), proving particularly effective for regularly shaped fruits. For instance, Lv et al. achieved fruit recognition by combining RGB color features with the Canny operator and Hough transform (Lv et al., 2015), while Tan et al. utilized Canny edge detection to extract edge features from apples, lemons, and mangoes for subsequent classification using machine learning (Tan et al., 2021). However, the robustness of these traditional methods is often limited in complex scenarios or when detecting occluded fruits. To enhance accuracy, Rabby et al. successfully implemented fruit recognition and classification in controlled background settings by integrating color and morphological features (Rabby et al., 2018). Furthermore, texture features, including but not limited to those derived from the Gray-Level Co-occurrence Matrix (GLCM) and Local Binary Patterns (LBP), play a crucial role in fruit object detection (Aygün and Güneş, 2017; Gurubelli et al., 2020).

Furthermore, Haar-like features (Besnassi et al., 2020) and Histogram of Oriented Gradients (HOG) features (Zhou and Yu, 2021) are also widely employed for image description and fruit recognition. Haar-like features extract discriminative information by computing differences in pixel intensities within rectangular regions. While achieving notable success in facial recognition, this approach has also been effectively applied to fruit detection within the agricultural domain. Conversely, HOG features facilitate classifier recognition of fruits by quantifying the distribution of gradient orientations within localized image regions.

With the advancement of machine learning technologies, traditional methods have progressively been integrated with machine learning classifiers, forming feature-based + classifier frameworks for object detection. These classifiers encompass Support Vector Machines (SVM), Random Forests (RF), KNearest Neighbors (KNN), and Naïve Bayes, among others. For instance, Zhang et al. (Zhang and Wu, 2012) achieved the classification of multiple fruit types using an SVM, achieving an accuracy of 88.2%, while Lin et al. successfully identified six fruit types employing the Hough transform combined with an SVM (Lin et al., 2020). RF enhances classification stability by aggregating predictions from multiple decision trees (Ramisetty et al., 2022), whereas KNN classifies fruits such as apples and dragon fruit based on sample similarity (Aulia et al., 2023). Naïve Bayes performs well in relatively straightforward classification scenarios, demonstrating effectiveness in non-destructive testing applications for apples (Miriti, 2016; Yogesh et al., 2021).

Prior to the widespread adoption of deep learning, methods based on handcrafted features and machine learning classifiers constituted the mainstream approach in object detection. Although demonstrating satisfactory performance in simple scenarios, their heavy reliance on manually designed features resulted in suboptimal effectiveness when confronted with complex environments. However, the rise of deep learning has precipitated a paradigm shift, with automated feature learning progressively supplanting handcrafted feature engineering to become the dominant technology in object detection.

### 3.2 Object detection technology based on deep learning

Driven by the advancement of agricultural automation and intelligence, the application of deep learning technologies in fruit harvesting has emerged as a prominent research focus. Fruit harvesting confronts multiple challenges, including object recognition in complex environments, identification and localization of diverse fruit types, maturity assessment, and occlusion handling. Traditional manual or mechanical methods are often characterized by low efficiency, high costs, and significant environmental constraints. In contrast, deep learning techniques, particularly Convolutional Neural Networks (CNNs) and their extensions such as Faster R-CNN, DETR, and YOLO, have significantly propelled the intelligence and automation of fruit harvesting robots.

#### 3.2.1 Two-stage object detection methods

Early object detection methods primarily relied on traditional CNN architectures like LeNet and AlexNet. While successful in image classification tasks, these networks inherently lacked the capability to directly output positional information. To address this limitation, the R-CNN approach proposed by Ross et al. pioneered the two-stage object detection paradigm by combining region proposal generation with deep feature extraction (Girshick, 2015). Subsequent advancements, namely Fast R-CNN and Faster R-CNN, substantially improved detection speed and accuracy through shared convolutional feature maps and the introduction of a Region Proposal Network (RPN) (Ren et al., 2016). The Feature Pyramid Network (FPN) further optimized Faster R-CNN by constructing a pyramid structure on feature maps of different scales, thereby enhancing multi-scale object detection capabilities (Lin et al., 2017). For example, Wan et al. achieved multiclass fruit detection using Faster R-CNN (Wan and Goudos, 2020), while Parvathi et al. applied Faster R-CNN for the detection of coconut maturity in complex backgrounds (Parvathi and Selvi, 2021).

Mask R-CNN is based on Faster R-CNN and achieves precise segmentation and localization of each instance object by adding pixel-level masks (He et al., 2017). This method has been applied to the identification of pick-up points, such as López-Barrios et al. (2023) who used Mask R-CNN to detect green bell peppers in greenhouses, successfully locating pick-up points. Despite the accuracy advantage of two-stage networks, they are computationally expensive and slow. Therefore, with the increasing demand for real-time performance, researchers have gradually shifted toward more efficient one-stage object detection methods.

#### 3.2.2 One-stage object detection methods

The YOLO (You Only Look Once) family represents a milestone in one-stage object detection models by transforming object localization into a regression problem through a fully convolutional architecture, achieving high detection speed (Redmon, 2016). With successive iterations, YOLO models have steadily improved in both accuracy and efficiency. Among earlier versions, YOLOv5 gained widespread adoption in agricultural scenarios due to its streamlined architecture and training efficiency (Wang et al., 2022; Hou et al., 2022). For instance, Sozzi et al. (2022) validated YOLOv5’s reliability in grape cluster detection across YOLOv3, YOLOv4, and YOLOv5 models.

Recent versions have introduced more advanced designs tailored for real-time and complex environments. YOLOv6 incorporates cross-layer feature fusion strategies to enhance real-time performance in industrial contexts (Li C. et al., 2022), while YOLOv8 significantly improves multi-scale object detection and feature extraction (Hussain, 2024). In agricultural applications, Wang et al. (2025) proposed a customized YOLO-ALW model based on YOLOv8, achieving 99.1% mAP in pepper detection tasks.

**Figure 6:**
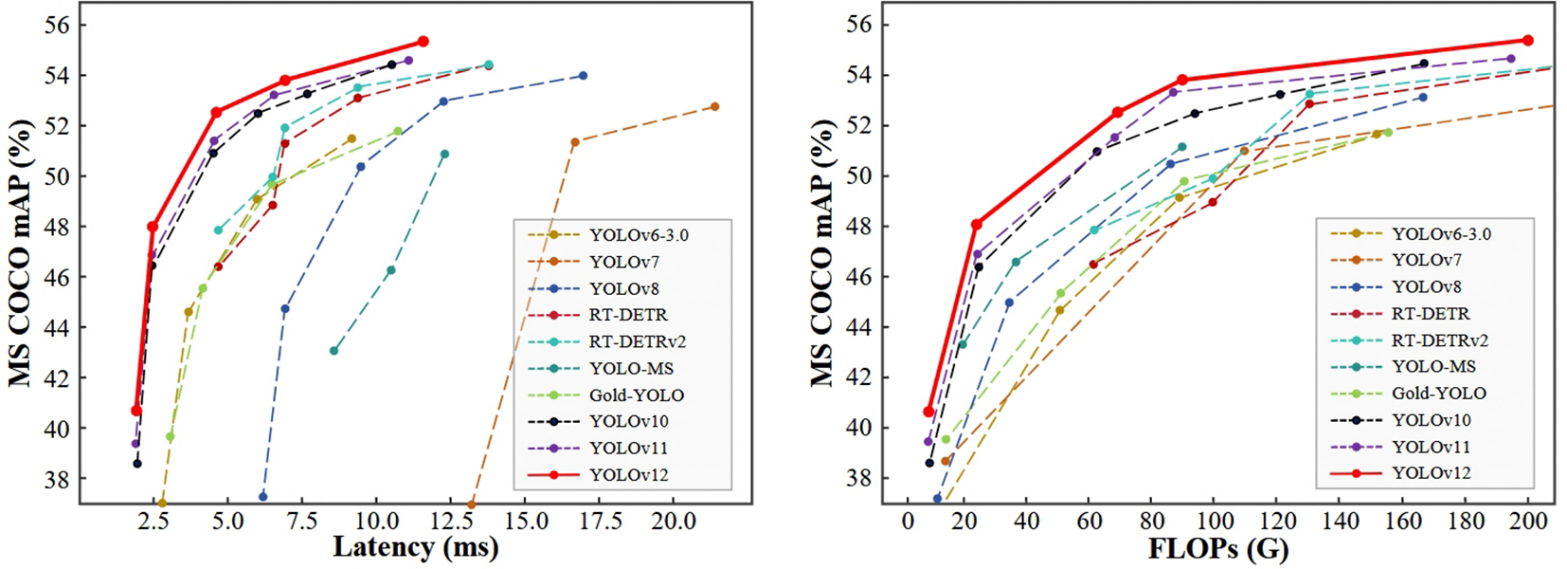
Performance comparison chart of YOLO series.

Further developments from YOLOv9 to YOLOv12 introduced architectural innovations such as reversible branches, the GELAN backbone, and modules like C2f-faster and Area Attention, improving detection precision while reducing inference latency (Khanam and Hussain, 2024; Wang A. et al., 2024; Wang CY. et al., 2024; Tian et al., 2025). **Figure 6** presents a comparison of latency (left) and computational complexity (FLOPs, right) against mAP on the MS COCO dataset. YOLOv12 achieves superior mAP while maintaining low latency and FLOPs, demonstrating outstanding overall efficiency. However, Sapkota et al. conducted a comprehensive evaluation of YOLOv8 through YOLOv12 in complex orchard environments and found that YOLOv9 delivered the best performance for green apple detection and counting (Sapkota and Karkee, 2025). Most recently, YOLOv13 introduced HyperACE (Hypergraph Adaptive Correlation Enhancement) and the FullPAD mechanism, further boosting detection performance (Lei et al., 2025). These advances suggest strong potential for future application in intelligent fruit harvesting.

In summary, while newer YOLO variants offer enhanced accuracy and speed, their effectiveness in agricultural environments depends on task-specific factors such as target size, occlusion level, and real-time requirements. Selecting the most suitable version requires careful consideration of these variables.

#### 3.2.3 Transformer-based object detection methods

Originally achieving remarkable success in natural language processing, Transformer architectures have recently been introduced into the field of object detection due to their ability to model global dependencies via self-attention mechanisms. Representative models include DETR (Zhao et al., 2024), Deformable DETR (Zhu et al., 2020), Swin Transformer (Liu et al., 2022), and Vision Transformer (ViT) (Huang et al., 2022). Compared with convolutional neural networks (CNNs), Transformer-based models enable end-to-end training without relying on predefined anchor boxes and offer strong global modeling capabilities, making them particularly suitable for complex agricultural environments with background clutter or occlusion.

Despite these advantages, Transformers still face several challenges in practical applications, including high computational cost, slow convergence, and a strong dependence on large-scale labeled datasets. To address these limitations, Guo et al. proposed a Transformer-based fruit detection framework, which effectively captures long-range dependencies but still struggles with tasks such as small object detection and fruit localization at boundaries (Guo et al., 2024).

To provide a comparative view of detection performance across different fruit types and detection models, **Tables 2**, **3** summarize the results reported in recent studies. “Results” refers to the reported detection accuracy under specific datasets or field conditions, while “Cycle Time” indicates the average time to complete a full picking cycle for each fruit, including perception, motion planning and execution, and fruit placement. These comparisons help illustrate the trade-offs between detection performance and overall harvesting efficiency across various algorithms and application contexts. To balance real-time performance and accuracy, recent research has begun to explore hybrid models that integrate Transformer modules into YOLO frameworks. Additionally, fusing Transformer features with multi-modal sensor data—such as RGB-Depth or thermal imagery—has emerged as a promising direction for enhancing robustness and accuracy in agricultural detection tasks.

**Table 2:** Part 1 of the research progress on various fruit harvesting visual perception technologies.

Fruit types	Technical solution	Results	Cycle time
Strawberry (Tafuro et al., 2022)	Detectron-2	AP50 = 94.19%	/
Tomato (Wu et al., 2021)	Stereo matching algorithm	/	13.2s
Grape (Luo et al., 2016)	Binocular stereo vision algorithm	Detection accuracy=87%	/
Coconuts (Parvathi and Selvi, 2021)	Improved Faster R-CNN with ResNet-50	mAP50 = 89.4%	/
Lychee (Guo et al., 2019)	Based on the CLAHE and Hough circle methods	F1 = 87.07%	/
Grape (Sozzi et al., 2022)	YOLOv3, YOLOv4, YOLOv5	F1 = 77%	/
Green Pepper (Wang F. et al., 2022)	YOLOv5s-CFL	mAP=95.46%	/
Lychee (Zhong et al., 2021)	MFBB	F1 = 83.8%	/
Citrus (Hou et al., 2022)	Improved YOLOv5s	F1 = 98.0%	/
Citrus (Li C. et al., 2023)	YOLOv5-CBAM	F1 = 92.41%	/
Zanthoxylum (Guo et al., 2023)	CA-DCNv2-YOLOv5	mAP=69.5%	/
Tomato (Chen W. et al., 2024)	YOLO-DNA	mAP=74%	/
Apple (Li H. et al., 2023)	BTC-YOLOv5s	mAP=84.3%	/
Green pepper (Huang et al., 2024)	Pepper-YOLO	mAP50 = 88.1%	/

**Table 3:** Part 2 of the research progress on various fruit harvesting visual perception technologies.

Fruit types	Technical solution	Results	Cycle time
Strawberry (Yu et al., 2020)	R-YOLO	recognition rate=94.43%	/
Grape (Chen J. et al., 2024)	YOLOv8-GP	mAP=89.7%	/
Longan (Chen et al., 2025)	Improved YOLOv8n	AP50 = 74.3%	/
Mango (Li et al., 2024)	Improved YOLOv8	mPA=84.9%	/
Strawberry (Xia, 2024)	Improved YOLOv8-Pose	mAP-kp=97.85%	/
Tomato (Liu et al., 2020)	YOLO-Tomato	AP=96.4%	/
Tomato (Lawal MO., 2021)	YOLO-Tomato-B	AP=99.3%	/
Green Sweet Pepper (Lopez-Barrios´ et al., 2023)	Mask R-CNN	mAP50 = 72.64%	/
Mango (Zheng et al., 2021)	Mask R-CNN	AP=82.4%	/
Strawberry (Mia et al., 2023)	DANet	mAP=78.27%	/
Tomato (Lawal OM., 2021)	YOLOMixNet	AP=98.4%	/
Apple (Li et al., 2023b)	MARL	Detection accuracy:71.28%-80.45%	5.8-6.7s
Lotus (Lu et al., 2024)	Three-view depth visual positioning method	Detection accuracy=98%	/
Sweet Pepper (Ning et al., 2022)	AYDY	Picking Rate=90.04%	/

## 4 Data labeling methods and localization techniques for fruit picking

The localization of picking points determines whether the fruit can be successfully harvested, making it one of the core aspects of the fruit picking process. In recent years, many scholars have focused on the labeling and research of fruit picking points. The methods for data labeling of fruit picking points and their localization and recognition are crucial elements in the research of intelligent harvesting robots. The goal is to ensure the accurate identification and localization of picking points through efficient and precise labeling methods and localization technologies, thereby enhancing the automation and intelligence of the harvesting machinery.

Selective picking methods are classified into two categories based on the way the fruit is harvested: picking the fruit itself and picking the fruit stem. The terminal operation methods differ between these two categories, and there are also significant differences in data labeling approaches. In recent years, many researchers have noted variations in the labeling of data for picking the same type of fruit, and these differences affect the picking accuracy.

**Figure 7:**
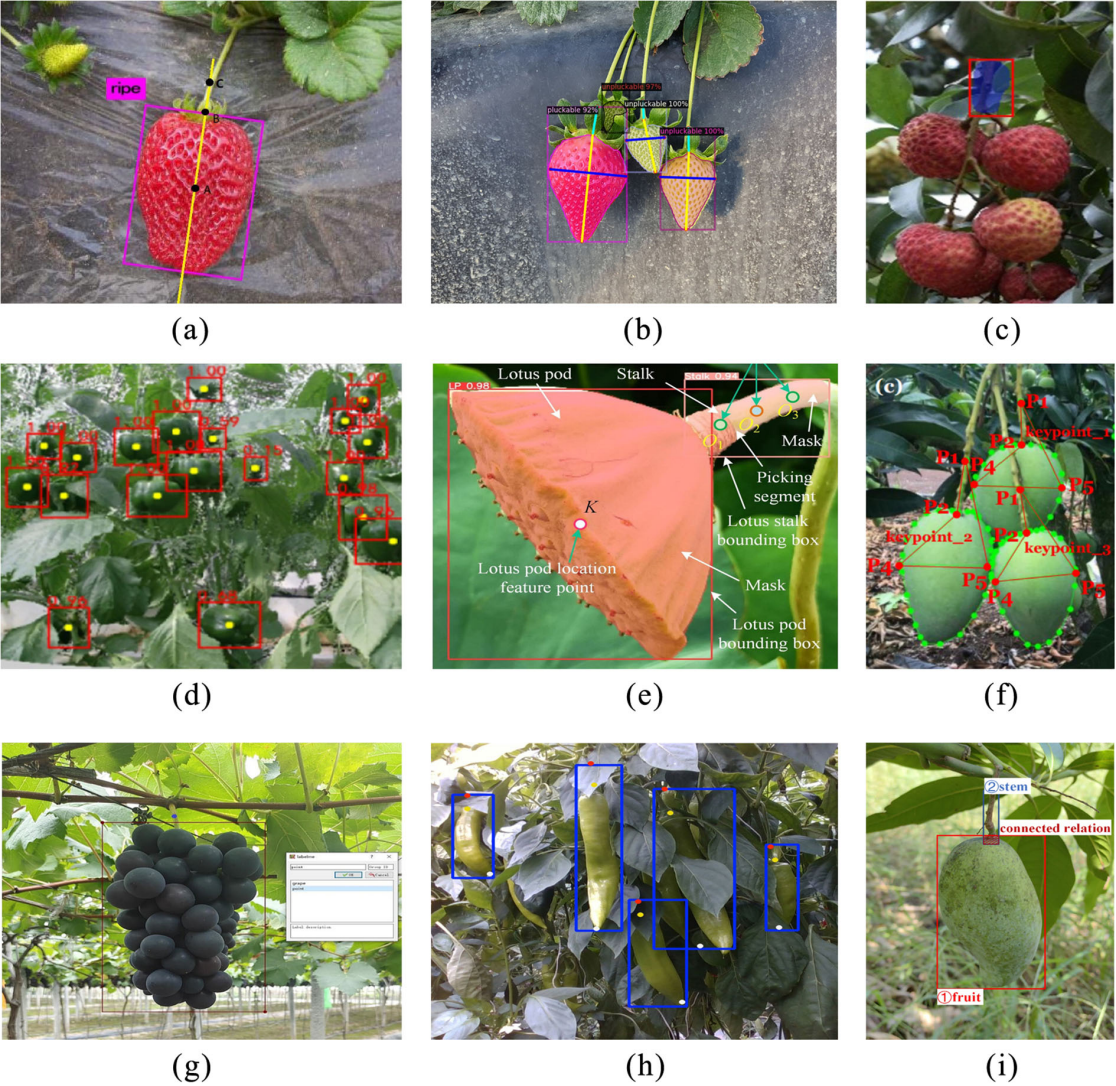
Labeling method for picking points of different fruits. **(a)** Strawberry picking point calculation (Yu et al., 2020), **(b)** Strawberry picking point calculation (Tafuro et al., 2022), **(c)** Litchi picking point calculation (Zhong et al., 2021), **(d)** Sweet pepper picking point (Ning et al., 2022), **(e)** Viburnum picking point calculation (Lu et al., 2024), **(f)** Mango picking point calculation (Zheng et al., 2021), **(g)** Grape picking point calculation (Chen W. et al., 2024), **(h)** Pepper picking point calculation (Huang et al., 2024), **(i)** Mango picking point calculation (Li et al., 2024).

Wang et al. applied prior knowledge of apples and used the Hough transform method and contour curvature to propose a method for calculating the contours of occluded apples to enable picking localization (Wang et al., 2016). This method struggles to identify the fruit when they overlap. Yu et al. labeled the strawberry body with a bounding box and used R-YOLO to predict the rotational boundaries of the strawberry and the physical size estimation of the picking point based on the strawberry’s rotation angle to confirm the picking point (Yu et al., 2020), as shown in **Figure 7a**. Tafuro used instance segmentation to label the strawberry body and calculated the fruit stem position and picking point localization by recognizing the boundary of the strawberry (**Figure 7b**) (Tafuro et al., 2022). Zhong et al. in their lychee picking labeling, only labeled the main fruit branch and took the center point of the bounding box as the picking point (Zhong et al., 2021), as shown in **Figure 7c**. If the center point is not exactly on the branch or is blocked by leaves, it can cause significant errors. **Figure 7d** shows the sweet pepper picking labeling, where both the bounding box and the center point of the fruit are estimated to confirm the picking position (Ning et al., 2022).

Lu et al. in their lotus pod picking used YOLOv5-based instance segmentation to label both the fruit region and the fruit stem region separately, and then calculated the key points from the segmented regions, inferring the picking position from those key points, as shown in **Figure 7e** (Lu et al., 2024). These various methods show the diversity in approaches to fruit picking point labeling and localization across different fruit types. The key challenge lies in ensuring high accuracy despite differences in fruit shapes, growth environments, and occlusions.

With the development of deep learning technologies, some researchers have shifted the fruit picking point localization from traditional geometric computations to regression-based calculations. Zheng et al. (2021) applied a combination of fruit instance segmentation and key point labeling for mango picking point localization, as shown in **Figure 7f**. They used the Mask RCNN model to simultaneously perform regression on the instance regions and multiple key points, with the picking point location ultimately determined by the key points. Chen et al (Chen W. et al., 2024), in their grape picking labeling work, used a fruit target bounding box and a fruit stem picking key point to label the data, and directly applied the YOLOv8pose model for regression calculations to achieve picking point localization, as shown in **Figure 7g**. To address the issue of chili picking points being occluded in complex scenarios, Huang et al. (2024) improved the YOLOv8-pose model by introducing a reversible network structure and a feature fusion module to achieve the recognition of multiple key points of the chili. The precise estimation of the picking points is realized through these key points, with the detection results shown in **Figure 7h**. Li et al (Li et al., 2024), in their mango picking work, combined object detection and instance segmentation. They first used two target bounding boxes to separately label the mango body and fruit stem, then applied instance segmentation to label the fruit stem region. After detecting the fruit stem using object detection, they performed instance segmentation on the stem region to obtain the skeleton line of the fruit stem, which was then used to calculate the picking point, as shown in **Figure 7i**.

In summary, we can observe that in recent years, there have been multiple labeling methods and picking point calculation approaches for the same fruit or different fruits with similar picking methods. The accuracy of the models trained or computed with different labeling methods also varies. In complex environments, how to develop a fruit labeling method that serves fruit picking tasks becomes particularly crucial. One of the key challenges in fruit picking work has always been how to minimize the position error of the fruit picking points.

## 5 Robot mobility and global environment perception technologies

### 5.1 Visual perception and navigation

Visual perception is one of the core technologies enabling fruit harvesting robots to achieve autonomous navigation and environmental understanding. By integrating Visual Simultaneous Localization and Mapping (V-SLAM) systems, robots can construct 3D maps and localize themselves in complex orchard environments, thereby enhancing their autonomous navigation capabilities. Chen et al. (2021) proposed a framework combining eye-in-hand stereo vision with SLAM, addressing the limitations of traditional SLAM methods in orchard environments and providing a solution for large-scale orchard harvesting that adapts to complex terrain and varying lighting conditions. Maud et al. (2023) utilized object detection and RTAB-Map algorithms to propose a real-time 3D mapping and localization system, optimizing the detection and management of palm oil trees and improving tree localization accuracy in large-scale plantations. Wang P. et al. (2025) based their approach on visual SLAM combined with semantic segmentation networks, improving the representation of point clouds and enhancing real-time processing speed, thus enabling more precise navigation and perception in greenhouse environments. These studies show that the combination of stereo vision with SLAM, particularly with the introduction of semantic SLAM, significantly enhances the robot’s perception and navigation accuracy in complex environments.

### 5.2 Path planning for mobile robots

Path planning is crucial for fruit harvesting robots to operate efficiently, particularly in complex orchard environments where optimizing paths to minimize time and energy consumption is essential. Urvina et al. (2024) proposed a combined global and local planning strategy, using the Traveling Salesman Problem (TSP) and the Informed Rapidly-exploring Random Tree (IRRT*) algorithm to optimize paths and avoid obstacles, improving navigation efficiency in complex terrain. Wang L. et al. (2022) introduced a full-coverage path planning method based on multi-objective constraints, which enhances the adaptability of path planning algorithms in irregular terrains, ensuring complete coverage. Wang et al. (2025a). developed a hybrid path planning approach, combining inner spiral and improved nested methods, significantly reducing non-work path length and improving operational coverage. These studies highlight the progression of path planning technologies toward combining global and local strategies, addressing path optimization challenges in complex agricultural environments.

### 5.3 Task scheduling

Task scheduling is vital for enhancing the efficiency of multi-tasking harvesting robots, especially when multiple tasks are performed simultaneously. Efficient task allocation and resource optimization are key to improving robot performance. Li et al. (2023a) proposed a Multi-Agent Reinforcement Learning (MARL)based scheduling method that dynamically adjusts task allocation based on real-time environment changes and task priorities, boosting operational efficiency. Wang et al. (2025b) addressed collaborative scheduling between harvesters and transport robots, introducing a task allocation and path planning method based on topological maps, significantly enhancing operational efficiency. Zhu et al. (2025) developed a task scheduling method for dual-arm robots using Mixed-Integer Linear Programming (MILP), optimizing task coordination and substantially improving strawberry harvesting throughput. These studies demonstrate that incorporating multi-agent systems and optimization algorithms into task scheduling can effectively enhance multi-task coordination and improve overall operational efficiency.

## 6 Optimal viewpoint planning for fruit picking

During the fruit picking process, environmental factors such as exposure, backlighting, shadows, occlusions, and vibrations may cause changes in the fruit’s position or lead to recognition failures. These factors not only result in the loss of visual information but may also prevent the accurate localization of picking points, ultimately reducing picking efficiency (Suresh Kumar and Mohan, 2023). For example, under strong sunlight or backlighting conditions, the camera may fail to clearly capture the fruit’s outline, while shadowed areas may obscure parts of the fruit, causing recognition errors. Vibration or mechanical movement can also shift the fruit’s position in the visual sensor, further affecting the accuracy and efficiency of the picking task. In addition, different viewpoints may produce varying picking outcomes. To address these issues, viewpoint planning, as an important technical measure, aims to maximize the fruit’s visibility and recognition rate by selecting the most appropriate angle, thereby minimizing the impact of external factors on recognition effectiveness (Yi et al., 2024). Viewpoint planning for fruit picking can be divided into four types based on the methods used: geometry-based viewpoint planning, information-based viewpoint planning, optimization-based viewpoint planning, and learning-based viewpoint planning.

### 6.1 Geometry-based viewpoint planning method

**Figure 8:**
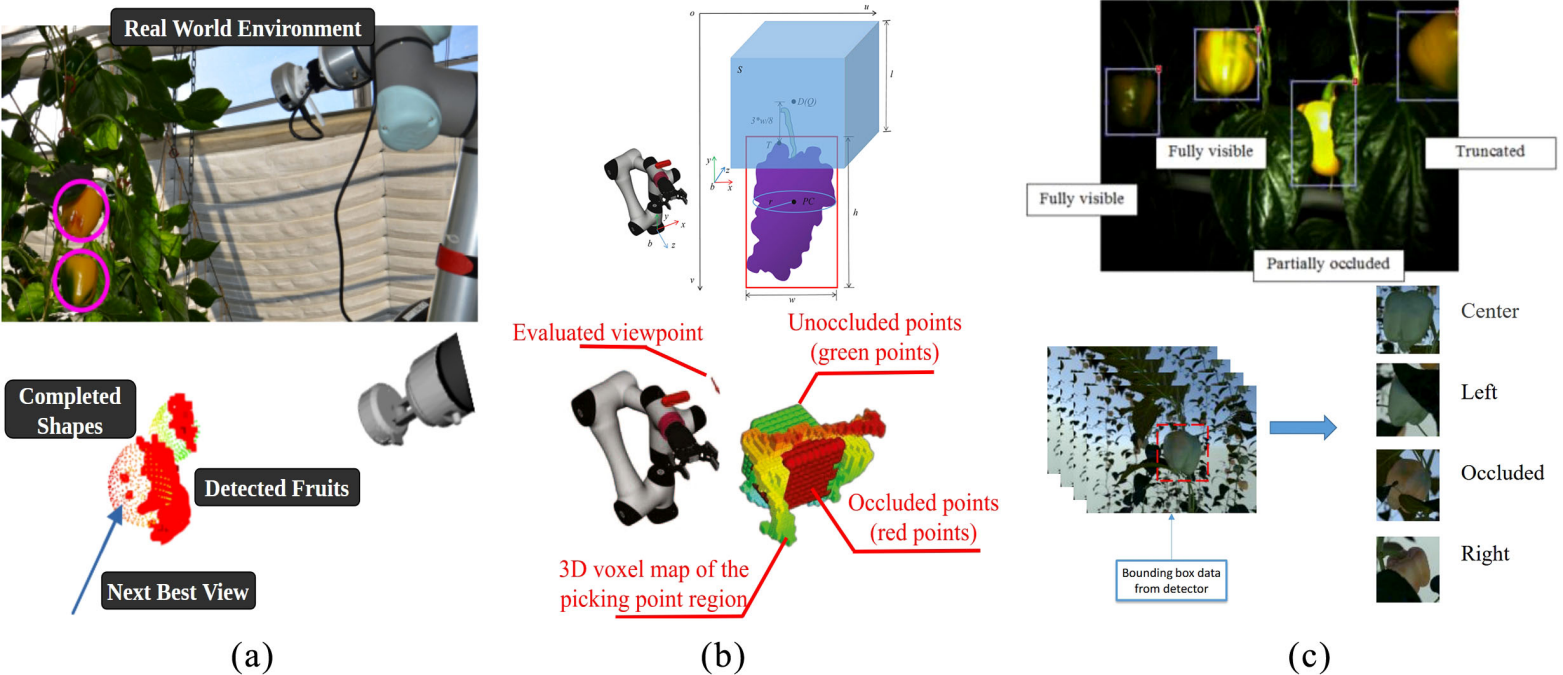
Different methods for calculating the optimal viewpoint. **(a)** Evaluate the picking point location through fruit shape completion, **(b)** Calculate the unobstructed areas of grape picking points using a scoring function, **(c)** Identify the optimal viewpoint through deep learning.

The geometric-based viewpoint planning method focuses on selecting the optimal viewpoint by calculating the spatial relationships between the environment and the target object. It typically involves using depth cameras or LiDAR to create an environmental model, which includes geometric shapes such as tree structures, fruit positions, and the locations of branches and leaves. The visual system then identifies the position of the target fruit and analyzes the feasibility of viewpoint selection based on the geometric relationship between the fruit and the environment. Once the best viewpoint is selected, it notifies the robotic arm to carry out the picking task. Menon et al. planned the optimal picking viewpoint based on the completeness of the fruit’s shape, as shown in **Figure 8a** (Menon et al., 2023). Hornung et al. proposed a 3D point cloud mapping based on octrees to simulate the robot’s 3D environment (Hornung et al., 2013). RVP constructed a voxel map of the fruit region and used a utility function based on expected information of the fruit region to evaluate candidate viewpoints (Zaenker et al., 2021). Burusa et al. drove next-best-view (NBV) planning through the tomato plant’s structural features and an attention mechanism (Burusa et al., 2024).

These methods have high computational complexity, are heavily dependent on equipment, and may become ineffective if the environment changes, such as when leaves or fruits sway, making pre-computed optimal viewpoints unsuitable.

### 6.2 Information-based and optimization-based viewpoint planning methods

Information-based and optimization-based viewpoint planning methods evaluate the characteristics of different viewpoints to select the ones that provide the maximum perceptual information or optimize task execution. These methods are widely applied in complex scenarios, such as fruit harvesting tasks. Yi et al. generated viewpoints randomly and guided the robotic arm to adjust its perspective by combining spatial coverage and motion cost to optimize the scoring function, as shown in **Figure 8b** (Yi et al., 2024); Menon et al. estimated missing information through shape completion and used an NBV-SC planner to find the best viewpoint (Menon et al., 2023); Akshay et al. made multi-viewpoint semantic perception decisions to determine the best viewpoint in tomato harvesting, achieving better results than active vision strategies (Burusa et al., 2024); Zaenker et al. designed a viewpoint motion planner to optimize the information gain for pepper detection (Zaenker et al., 2023). These methods require evaluating multiple viewpoints, resulting in a large computational load that affects real-time performance. Optimization-based viewpoint planning, on the other hand, uses optimization algorithms to select viewpoints, with objectives typically focused on minimizing occlusion, maximizing information gain, or improving task efficiency. These methods evaluate the quality of viewpoints by setting objective functions. For example, Li et al. improved YOLOv5 and combined it with the ant colony algorithm to optimize the harvesting sequence of citrus, addressing collision issues (Li C. et al., 2023); Li et al. used reinforcement learning to define a reward function for optimizing harvesting strategies in a multi-arm system (Li et al., 2023b); Yi et al. generated candidate viewpoints and scored them to select the best perspective (Yi et al., 2024). Optimization-based methods also require evaluating multiple viewpoints, which imposes a large computational burden, especially in large-scale and dynamic environments, affecting real-time performance.

### 6.3 Learning-based viewpoint planning methods

Learning-based planning methods utilize machine learning and deep learning techniques to train models that learn how to select the optimal viewpoint based on occlusion conditions. These methods offer high adaptability and flexibility, performing particularly well in complex and dynamic environments. Learning-based viewpoint planning works by automatically extracting features from a large amount of training data and making predictions using learned models. The models can include deep neural networks, reinforcement learning models, and others. The learning process typically involves using historical data to train the model, enabling it to generate reasonable viewpoint selection strategies based on input environmental information or task requirements. Zhang et al. applied deep learning techniques for multiview fruit detection in apple picking to determine the optimal picking location (Zhang et al., 2022). Wang et al. used a few-shot reinforcement learning approach to jointly train the Next Best View (NBV) and Next Best Point (NBP), with the model continuously optimizing viewpoint decisions through interaction with the environment (Wang G. et al., 2024). Chen et al. employed YOLOv8 for real-time object detection of longan fruits and guided a drone to perform fruit picking by establishing the relationship between the target points and the drone’s speed (Chen et al., 2025). Rehman et al. conducted viewpoint data collection by rotating 30 degrees from left to right around the target in a nighttime environment, using deep learning techniques to identify occluded areas and guide the harvesting robot in selecting the optimal viewpoint, as shown in **Figure 8c** (Rehman and Miura, 2021).

Overall, with the enhancement of perception and computational capabilities, significant progress has been made in fruit harvesting viewpoint planning technology. Geometric, information-based, optimization, and learning methods each have their advantages, adapting to different scenarios and requirements. Geometric methods are precise but complex and dependent on specific conditions; information-based methods optimize viewpoints but are computationally intensive; optimization methods are effective but burdensome in complex environments; and learning methods are highly adaptable but rely on training data and resources. Although existing research has improved recognition and harvesting efficiency, real-time performance, robustness, and accuracy in complex environments remain major challenges. Future research could explore the integration of multiple methods, such as combining optimization with deep learning, to enhance efficiency, reduce computational consumption, and improve the system’s adaptability and real-time adjustment capabilities.

## 7 Discussion

The fruit-picking robot has made significant advancements in visual perception technology, which is central to the automation of fruit harvesting. However, despite continuous technological progress, there are still many challenges when it comes to applying these systems in real agricultural environments.

### 7.1 Technical challenges and limitations

Various advanced cameras, such as monocular, binocular, and 3D depth sensors, have enhanced the precision of fruit recognition and localization for robots. Binocular cameras provide depth information through disparity, but they have limitations in calibration and adaptability. Complex depth sensors, such as Time-of-Flight (ToF) cameras and structured light cameras, offer excellent depth perception but are expensive and computationally intensive. Deep learning algorithms, such as YOLO, have improved fruit detection accuracy, but they require powerful computational resources, large training datasets, and depth data fusion. Striking a balance between computational efficiency and accuracy remains a key challenge for large-scale applications.

### 7.2 Impact of environmental variations

Intelligent fruit-picking robots face challenges such as lighting variations, plant positioning, and fruit occlusion in agricultural environments. These factors complicate the visual system’s ability to detect and localize fruits accurately. Even advanced sensors struggle when confronted with real-world agricultural settings. For instance, differences in the shape, color, and growth patterns of various fruits increase the difficulty of segmentation and classification. Ensuring high-precision recognition amidst these variations remains an unsolved problem.

### 7.3 Picking accuracy and efficiency

Picking accuracy is crucial, particularly in minimizing damage and improving fruit quality. Visual reconstruction and depth perception technologies assist in pinpointing the picking location, but the high computational cost remains a bottleneck in real-time data processing. Enhancing operational precision and preventing fruit damage are key considerations. Additionally, the introduction of active vision technology, which adjusts the visual angle based on real-time perception, can further improve picking accuracy.

### 7.4 Future development directions

Despite the challenges, the future of intelligent fruit-picking robots remains promising. Future research could explore sensor fusion, integrating visual, tactile, and force data to enhance the robot’s overall environmental perception. AI and machine learning, particularly unsupervised learning, hold the potential to reduce the reliance on large labeled datasets and improve the robot’s adaptability to new environments. By combining deep learning-based visual servoing techniques, path planning, and control strategies can be optimized. In the future, intelligent fruit-picking robots will achieve a better balance between real-time performance and accuracy.

## 8 Conclusion

In this paper, we reviewed the research progress of visual perception technology in intelligent fruit-picking robots. First, we introduced the advantages and disadvantages of different types of cameras: monocular cameras are suitable for simple scenarios, binocular cameras provide depth information for moderately complex environments, while structured light and ToF depth cameras perform excellently in high-precision depth perception and complex environments.

Next, we explored the application of object detection technology in fruit picking, comparing traditional image processing methods with modern deep learning methods such as YOLO and SSD. While deep learning methods offer higher accuracy and better adaptability, they require large amounts of training data and high-performance hardware. Traditional methods still have advantages when resources are limited.

Regarding the localization of picking points, we reviewed vision-based 3D reconstruction and depth perception methods, emphasizing the importance of accurate localization to improve the picking success rate and reduce fruit damage. Additionally, we explored technologies such as V-SLAM, mobile path planning, and task scheduling, which contribute to enhancing the robot’s operational efficiency throughout the entire orchard. We also discussed the combination of active vision and visual servoing techniques, showing that these two technologies can significantly enhance the robot’s adaptability and precision in dynamic environments. By adjusting the visual angle in real-time and optimizing control strategies, robots can more accurately locate and manipulate targets, especially when dealing with fruit occlusion and complex backgrounds.

Finally, we summarized the current status and future development directions of visual perception technology. Despite significant progress, challenges such as poor environmental adaptability, low system integration, and high costs still exist in real agricultural environments. With the continuous development of computer vision, deep learning, and sensor technologies, the future intelligent fruit-picking robots, combining active vision and visual servoing techniques, will make greater breakthroughs in efficiency and accuracy and will be capable of addressing more complex application scenarios.
